# Obstructed Hemivagina and Ipsilateral Renal Agenesis Syndrome: A Case Report

**DOI:** 10.31729/jnma.7444

**Published:** 2022-06-30

**Authors:** Ashok Chapagain, Keshab Paudel, Sunil Mani Pokhrel, Ram Prasad Sapkota

**Affiliations:** 1 Department of Radiology and Imaging, Bharatpur Hospital, Chitwan, Nepal; 2 Department of Obstetrics and Gynaecology, Bharatpur Hospital, Chitwan, Nepal.

**Keywords:** *case reports*, *hematocolpos*, *mullerian ducts*, *unilateral renal agenesis*

## Abstract

Obstructed hemivagina and ipsilateral renal anomaly syndrome also known as Herlyn-Werner-Wunderlich syndrome is a rare congenital urogenital anomaly characterised by Mullerian duct anomalies associated with mesonephric duct anomalies. A 10-year old female presented with acute lower abdominal pain, urinary retention and scanty menstrual flow during her first menstruation. Ultrasonography and contrast computed tomography showed uterine didelphys, hematocolpos, obstructed hemivagina and left renal agenesis. Hemivaginal septal resection and drainage of the hematocolpos were done and operative findings also confirmed the final diagnosis. She was discharged and followed up after 2 weeks and her symptoms had resolved completely. Being a rare entity many clinicians and radiologists are unaware of this disease so this may lead to misdiagnosis whenever these cases present. So strong suspicion and knowledge of this disease entity are essential for a precise diagnosis.

## INTRODUCTION

Obstructed Hemivagina and Ipsilateral Renal Aanomaly (OHVIRA) is a rare congenital urogenital anomaly characterised by Mullerian Duct Anomalies (MDA) associated with mesonephric duct anomalies. This entity is also known as Herlyn-Werner-Wunderlich (HWW) syndrome. The incidence of HWW syndrome ranges from 1/2,000 to 1/28,000, and it is accompanied by unilateral renal agenesis in 43% of cases. Usually, it is diagnosed after menarche due to recurrent severe dysmenorrhea.^1^ This case report is important to emphasise that a high suspicion of OHVIRA syndrome should be there in a young female who has a finding of hematocolpos in the ultrasonography scan and so uterine anomaly and associated renal anomaly should be carefully looked upon at imaging.

## CASE REPORT

A 10-year-old girl presented to the emergency department with a history of scanty menstrual bleeding during her first menarche followed by lower abdominal pain and acute urinary retention. There was no history of any abnormal vaginal discharge in the past. There was no significant history of any genetic disorders in her family.

A general physical examination done at the emergency department only revealed a distended urinary bladder. Ultrasound of abdomen and pelvis was done from the emergency department which showed hematocolpos, widely separated double uteri and left renal agenesis. A provisional diagnosis of OHVIRA syndrome was made and advised for further diagnostic workup. The gynaecology department attended the case in the emergency department and on per speculum examination an obstructed vagina was noted which further pointed towards the possible diagnosis of OHVIRA syndrome considering both ultrasound and per speculum examination findings. Since Magnetic Resonance Imaging (MRI) was unavailable in the hospital, contrast Computed Tomography (CT) including a delayed scan was advised by the department of obstetrics and gynaecology for further evaluation of the case, as a contrast CT scan is considered the best modality to diagnose renal anomalies including duplicated kidney, crossed fused renal ectopia, etc. which are all associated with the OHVIRA syndrome.

**Figure 1:**
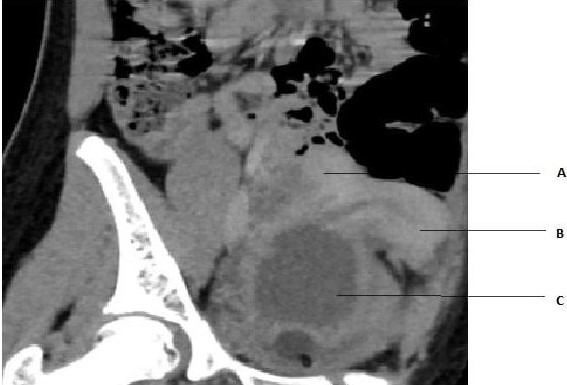
Contrast-enhanced CT scan image showing two separate uteri (A and B) with widely divergent apices, and two separate cervices. Left uterine and cervical cavities are seen in continuity with the hematocolpos (C).

The contrast CT showed two separate uteri (right and left) with widely divergent apices, two separate cervices, and high-density fluid collection in the vaginal cavity which was continuous with the left cervical and uterine cavity (Figure 1).

**Figure 2:**
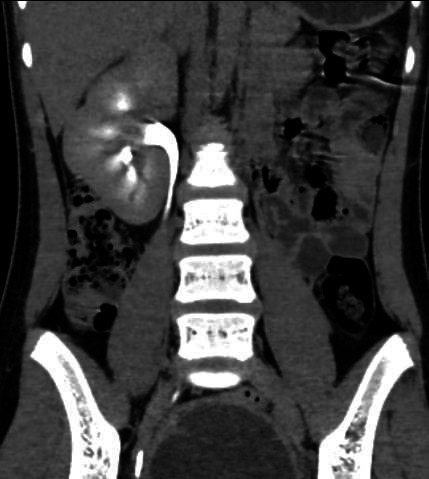
A delayed phase of contrast-enhanced CT scan of abdomen and pelvis showing associated left renal agenesis. The right kidney and pelvicalyceal system are normal.

Apart from this there was an absence of left kidney (Figure 2). No other renal anomalies were present. All other findings were normal in the CT scan.

Department of Gynaecology planned for vaginoplasty and drainage of hematoma as her definitive management. She was operated on the second day of her presentation and the operative finding after resection of obstructive vaginal septum revealed hematocolpos which was drained. After the drainage of the hematoma, a more clear view was obtained and it showed a single vaginal cavity leading into two cervical openings.

**Figure 3:**
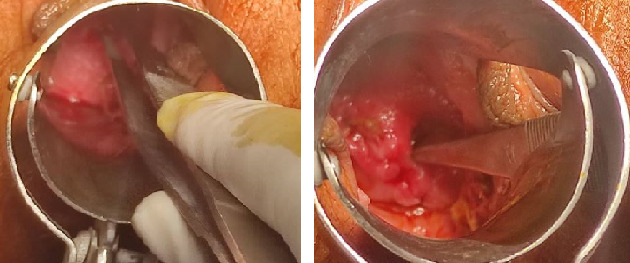
Postoperative per speculum finding shows two cervical openings, indicated by the tip of forceps, leading into a common vaginal cavity.

After the surgery, she was transferred to the gynaecology ward where she was treated with intravenous ceftriaxone 500 mg twice daily for 5 days, intravenous metronidazole 250 mg thrice daily for 5 days and local wound care. After a few days of her hospital stay, she was discharged and asked for follow up after two weeks or whenever necessary. She followed up after 2 weeks in the outpatient department and there were no complaints and her symptoms had completely resolved.

## DISCUSSION

Herlyn-Werner-Wunderlich (HWW) syndrome is characterised by a triad of Mullerian duct anomaly (uterus didelphys), obstructed hemivagina and mesonephric duct anomalies. Most often the mesonephric duct anomalies manifest as renal agenesis but duplicated kidneys, dysplastic kidneys, rectovesical bands or crossed fused ectopia may also be seen.^1^ The overall prevalence of Mullerian tract abnormality is 2% to 3%.^2^ The incidence of OHVIRA syndrome is still unknown, with only a few published case reports so far.^3^

The pathogenesis of OHVIRA syndrome is multifactorial and related to an anomaly in the development of paramesonephric (Mullerian) and mesonephric (Wolffian) ducts. The mesonephric ducts induce the normal development of Mullerian ducts and give origin to the kidneys. In OHVIRA syndrome abnormal development of the Mesonephric ducts leads to unilateral renal agenesis & since Mullerian ducts also fail to develop normally this will lead to uterine didelphys and imperforated hemivagina. As there is a close relationship between embryological development of the female urinary and reproductive symptoms, cases with renal malformations will benefit from the evaluation of the reproductive structures and vice versa.^4^

Given the association with a variety of renal tract anomalies, including ectopic ureters, consideration should be given to pre-operative renal tract imaging to prevent the risk of iatrogenic urinary incontinence.^5^ The first line of investigation in OHVIRA syndrome is an ultrasound scan, as it is readily available. However, MRI remains the imaging modality of choice to evaluate Mullerian duct abnormalities. MRI will accurately show the shape of the uterine cavity, contour, and cervical and vaginal abnormalities. Pre-operative imaging with MRI is essential to define the Mullerian duct structures and renal tract. It also assists with surgical planning.^6^ Laparoscopy is still the gold standard for the investigation of gynaecological congenital abnormalities but is only used if MRI fails to establish a diagnosis or MRI is unavailable.^7^ Contrast CT or CT Intravenous Urography is considered the best modality to rule out renal anomalies and thus may be required at times to diagnose the associated renal anomalies.^8^ Treatment of OHVIRA is vaginoplasty. Ideally, a single step surgery with drainage of the obstructed hemivagina and resection of the obstructing longitudinal vaginal septum is performed to restore normal vaginal function.^9^

There should be a high suspicion of OHVIRA syndrome when encountering adolescent female patients with nonspecific pelvic symptoms and unilateral renal agenesis since this disease won't present day to day and is an extremely rare entity. Radiologists and clinicians must have knowledge of this syndrome for early diagnosis and proper management to avoid misdiagnosis and mishaps in case management.
